# Prognostic Nomogram for Rectal Cancer Patients With Tumor Deposits

**DOI:** 10.3389/fonc.2022.808557

**Published:** 2022-02-02

**Authors:** Xiaohong Zhong, Lei Wang, Lingdong Shao, Xueqing Zhang, Liang Hong, Gang Chen, Junxin Wu

**Affiliations:** ^1^ Department of Radiation Oncology, Fujian Medical University Cancer Hospital, Fujian Cancer Hospital, Fuzhou, China; ^2^ Department of Pathology, Fujian Medical University Cancer Hospital, Fujian Cancer Hospital, Fuzhou, China; ^3^ Fujian Key Laboratory of Translational Cancer Medicine, Fuzhou, China

**Keywords:** rectal cancer, tumor deposits, nomogram, OS, SEER

## Abstract

**Aim:**

Tumor deposits (TDs) are an aggressive hallmark of rectal cancer, but their prognostic value has not been addressed in current staging systems. This study aimed to construct and validate a prognostic nomogram for rectal cancer patients with TDs.

**Methods:**

A total of 1,388 stage III–IV rectal cancer patients who underwent radical surgical resection from the Surveillance, Epidemiology, and End Results (SEER) database were retrospectively analyzed to identify the clinical value of TDs. TD-positive rectal cancer patients in the SEER database were used as the training set to construct a prognostic model, which was validated by Fujian Cancer Hospital. Three models were constructed to predict the prognosis of rectal cancer patients with TDs, including the least absolute shrinkage and selection operator regression (LASSO, model 1), backward stepwise regression (BSR, model 2), and LASSO followed by BSR (model 3). A nomogram was established among the three models.

**Results:**

In the entire cohort, TD was also identified as an independent risk factor for overall survival (OS), even after adjusting for baseline factors, stage, other risk factors, treatments, and all the included variables in this study (all *P* < 0.05). Among patients with TDs, model 3 exhibited a higher C-index and area under the curves (AUCs) at 3, 4, and 5 years compared with the American Joint Committee on Cancer staging system both in the training and validation sets (all *P* < 0.05). The nomogram obtained from model 3 showed good consistency based on the calibration curves and excellent clinical applicability by the decision curve analysis curves. In addition, patients were divided into two subgroups with apparently different OS according to the current nomogram (both *P* < 0.05), and only patients in the high-risk subgroup were found to benefit from postoperative radiotherapy (*P* < 0.05).

**Conclusion:**

We identified a novel nomogram that could not only predict the prognosis of rectal cancer patients with TDs but also provide reliable evidence for clinical decision-making.

## Introduction

Tumor deposits (TDs) were first reported in 1935 (1) and are associated with aggressive characteristics, advanced stage, and adverse prognosis of rectal cancers (2–4). The definition and origin of TD has always been disputed (3), although it was introduced in the fifth American Joint Committee on Cancer (AJCC) staging system for rectal cancer in 1997 (5). Until recently, TDs are defined as “irregular discrete tumor deposits in the perirectal fat that are away from the leading edge of the tumor and show no evidence of residual lymph node tissue, but that are within the lymphatic drainage of the primary tumor” in the latest edition of AJCC staging system (6). Considering that the detection rate of TDs is higher (4, 7), more concerns should be considered.

TDs are considered as an aggressive hallmark of rectal cancer not only in the absence of regional lymph node metastasis (LNM) (8, 9), but also in patients with LNM (10, 11). However, the clinical value of TDs is severely underestimated in the management of rectal cancer. TD-positive tumors are classified as N1c in the absence of LNM, while neither the presence nor the number of TDs is considered in the pN staging in cases of concomitant LNM (6). In addition, TD-positive patients have often been overlooked in the postoperative management of most of the current guidelines. As an efficient anti-recurrence prophylaxis and an alternative salvage strategy for recurrent tumors, postoperative radiotherapy (RT) is only recommended for N1c patients in the European Society for Medical Oncology (ESMO) clinical practice guideline for rectal cancer (12).

In the current study, we first identified the clinical significance of TDs in a population-based analysis of the Surveillance, Epidemiology, and End Results (SEER) database, and then constructed a nomogram to predict the prognosis of TD-positive rectal cancer patients, which was also validated by an external cohort from our center.

## Materials and Methods

### Ethics Statement

This study was conducted under the ethical guidelines of the Helsinki Declaration. We acquired approval from Fujian Cancer Hospital’s Ethics Committee (K2021-050-01), which waived back the individual informed consent owing to that the clinicopathological data were extracted retrospectively. On the other hand, we gained an official permit to access the research data from the SEER database.

### Study Design

**Figure 1:**
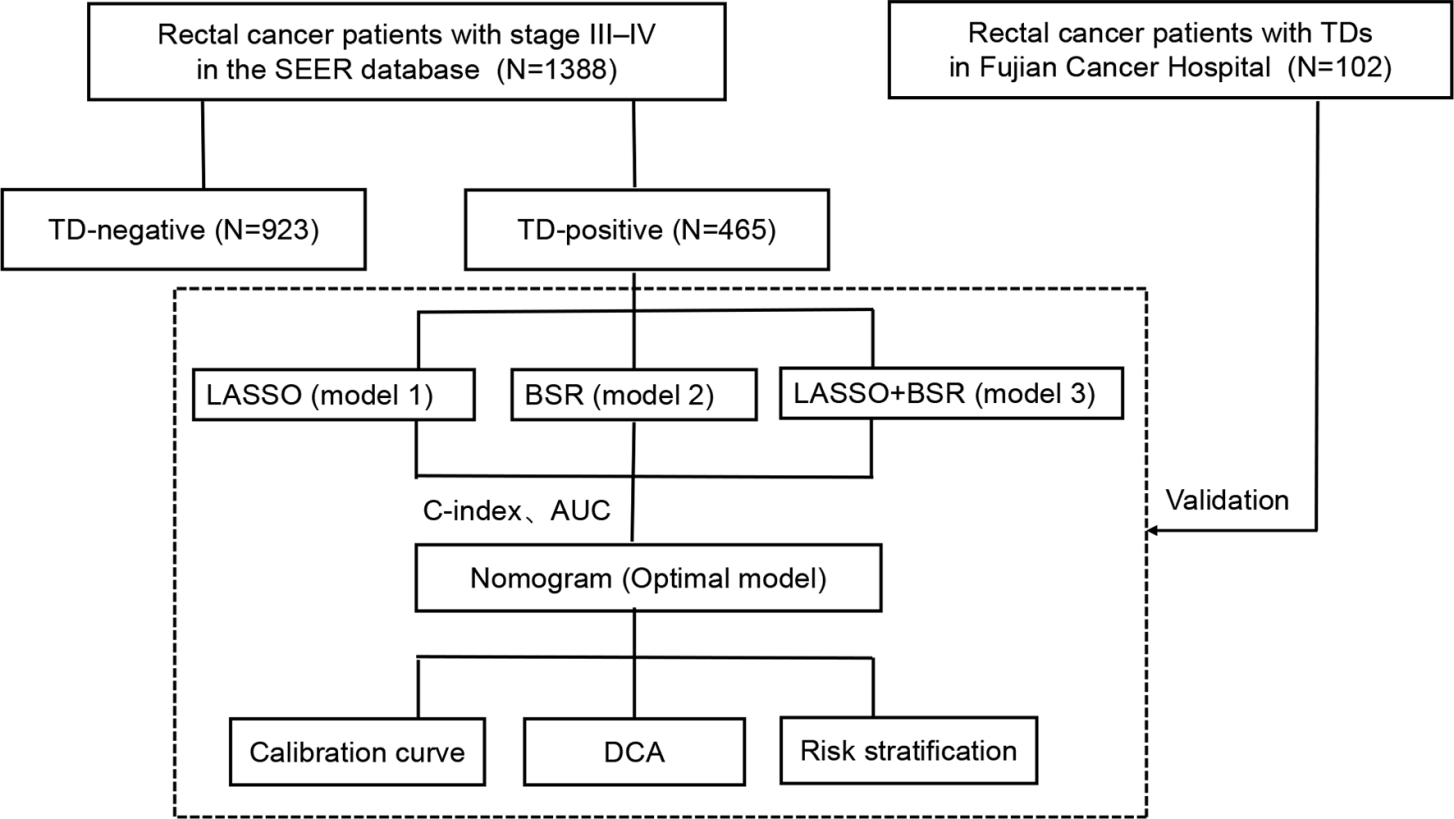
Flowchart of the study design. SEER, Surveillance, Epidemiology, and End Results; TD, tumor deposit; LASSO, least absolute shrinkage and selection operator regression; BSR, backward stepwise regression; C-index, concordance index; AUC, area under the curve; DCA, decision curve analysis.

**Figure 1** shows the flowchart of the current study. Stage III–IV rectal cancer patients who underwent radical surgical resection in the SEER database between January 2010 and December 2015 were studied, including age, sex, marital status, carcinoembryonic antigen (CEA) level, T stage, N stage, M stage, tumor size, tumor differentiation, perineural invasion (PNI) status, lymph node ratio (LNR), log odds of metastatic lymph nodes (LOODS), positive lymph node (PLNC), negative lymph node (NLNC), TD status, postoperative RT, postoperative chemotherapy, and follow-up. The exclusion criteria in this study were as follows: 1) underwent neoadjuvant therapy, 2) multiple primary cancers, and 3) T0/Tis.

First, we identified the clinical value of TDs in the entire cohort. TD-positive rectal cancer patients in the SEER database were used as the training set to construct a prognostic model. Data of rectal cancer patients with TDs from Fujian Cancer Hospital were used as an external cohort to verify the prognostic model.

### Clinicopathological Variable Stratification

Using the “surv_cutpoint” function from the “survminer” R package, LNR, LOODS, and PLNC were categorized as tritaxic variables with optimal cutoff values of 0.038 and 0.600, −1.330 and 0.160, and 0 and 4, while NLNC was classified as a dichotomous variable with an optimal cutoff value of 7. **Supplementary Figure 1** shows the excellent calibration of the current cutoff values of LNR, LOODS, PLNC, and NLNC in terms of overall survival (OS) (all *P* < 0.05).

Variables in this study were classified as follows: age at diagnosis (≤60 years, >60 years), sex (male or female), marital status (married, unmarried), CEA level (normal, elevated/borderline), stage (III, IV), T stage (T1–2, T3, T4), N stage (N1, N2), M stage (M0, M1), tumor size (≤5 cm, >5 cm), tumor differentiation (I/II and III/IV), PNI (absent, present), LNR (≤0.038, 0.038–0.600, >0.600), LOODS (≤−1.330, −1.330–0.160, >0.160), PLNC (0, 0–4, >4), NLNC (≤7, >7), postoperative RT (no, yes), postoperative chemotherapy (no, yes), and survival (months).

### Outcome Definition

The primary outcome measure for this study was OS, which was defined from the data obtained from the date of diagnosis through the date of either death or the latest follow-up.

### Variable Selection and Model Construction

To avoid underfitting and/or overfitting of the model, three advanced statistical methods, namely, the least absolute shrinkage and selection operator regression (LASSO, model 1) (13), backward stepwise regression (BSR, model 2) (14), and LASSO followed by BSR (model 3) (15), were adopted to screen the candidate variables in the training set. The optimal model was determined using Harrell’s concordance index (C-index) (16) and area under the curve (AUC) (17). All three models were compared with the eighth AJCC staging system.

### Performance and Validation of the Nomogram

A nomogram was derived using the optimal model. The discrimination of the nomogram was evaluated by the C-index and AUC as described above, and the predictive accuracy was assessed by the calibration curve (18). Decision curve analysis (DCA) (19) was performed to assess the potential clinical applicability and benefits of the nomogram. Similar analyses were conducted in the validation set.

The patients were divided into low-risk and high-risk groups according to the optimal cutoff value of the prognostic model risk score, which was determined by the “surv cutpoint” function from the “survminer” R package in the training set. Finally, we evaluated the effects of postoperative RT in different groups to screen patients who benefited from postoperative RT.

### Statistical Analyses

The Kaplan–Meier (K-M) method was used to compare OS among different groups using a log-rank test. LASSO and BSR were used to select variables. Multivariate Cox regression analysis was performed for model construction. Statistical tests were conducted using RStudio (version 1.3.1073), including xlsx, **Table 1**, survminer, survival, rms, nomogramFormula, timeROC, and stdca packages. All statistical tests were two-tailed, and statistical significance was set at *P <*0.05.

**Table 1:** Baseline clinicopathological characteristics and the status of tumor deposit in rectal cancer patients.

	Tumor deposit status		*P*-value
	Negative (*N* = 923)	Positive (*N* = 465)	
Age			
≤60 years	454 (49.2%)	231 (49.7%)	0.908
>60 years	469 (50.8%)	234 (50.3%)
Sex			
Male	507 (54.9%)	281 (60.4%)	0.058
Female	416 (45.1%)	184 (39.6%)
Marital status			
Unmarried	350 (37.9%)	206 (44.3%)	0.026
Married	573 (62.1%)	259 (55.7%)
Carcinoembryonic antigen			
Normal	521 (56.4%)	220 (47.3%)	0.002
Elevated/borderline	402 (43.6%)	245 (52.7%)
Stage			
III	759 (82.2%)	330 (71.0%)	<0.001
IV	164 (17.8%)	135 (29.0%)
T stage			
T1–2	266 (28.8%)	56 (12.1%)	<0.001
T3	571 (61.9%)	313 (67.3%)
T4	86 (9.3%)	96 (20.6%)
N stage			
N0	40 (4.3%)	0 (0%)	<0.001
N1	591 (64.0%)	241 (51.8%)
N2	292 (31.6%)	224 (48.2%)
M stage			
M0	759 (82.2%)	330 (71.0%)	<0.001
M1	164 (17.8%)	135 (29.0%)
Tumor size			
≤5 cm	616 (66.7%)	268 (57.6%)	0.001
>5 cm	307 (33.3%)	197 (42.4%)
Tumor differentiation			
Grade I/II	787 (85.3%)	347 (74.6%)	<0.001
Grade III/IV	136 (14.7%)	118 (25.4%)
Perineural invasion			
Absent	755 (81.8%)	279 (60.0%)	<0.001
Present	168 (18.2%)	186 (40.0%)
LNR			
≤0.038	93 (10.1%)	101 (21.7%)	<0.001
≤0.600	772 (83.6%)	286 (61.5%)
>0.600	58 (6.3%)	78 (16.8%)
LOODS			
≤−1.330	58 (6.3%)	78 (16.8%)	<0.001
≤0.160	802 (86.9%)	309 (66.4%)
>0.160	63 (6.8%)	78 (16.8%)
PLNC			
0	40 (4.4%)	84 (18.1%)	<0.001
≤4	674 (73.0%)	192 (41.3%)
>4	209 (22.6%)	189 (40.6%)
NLNC			
≤7	118 (12.8%)	112 (24.1%)	<0.001
>7	805 (87.2%)	353 (75.9%)
Postoperative radiotherapy			
No	535 (58.0%)	291 (62.6%)	0.110
Yes	388 (42.0%)	174 (37.4%)
Postoperative chemotherapy			
No	235 (25.5%)	125 (26.9%)	0.613
Yes	688 (74.5%)	340 (73.1%)

LND, dissected lymph nodes; LNR, lymph node ratio; LOODS, log odds of metastatic lymph nodes; PLN, positive lymph node; NLN, negative lymph node.

## Results

### Characteristics Comparison Between Patients With and Without TDs

A total of 1,338 patients were eligible for this study, including 465 (34.8%) patients with TDs. The clinicopathological characteristics of patients with and without TDs are depicted in **Table 1**. As expected, TD-positive patients typically present with aggressive characteristics, such as elevated CEA levels, advanced tumor-node-metastasis (TNM) stage, and PNI (all *P* < 0.05, **Table 1**). Notably, no significant differences were observed between patients with and without TDs in terms of receiving postoperative RT and postoperative chemotherapy (both *P* > 0.05, **Table 1**).

### Significance of TDs in Patients With Rectal Cancer

TDs were identified as a risk factor for OS using univariate Cox regression analysis (*P* < 0.001, **Table 2**). To decrease the potential confounding bias, adjusted hazard ratios (HRs) were adopted to determine the effect of TDs on the prognosis of rectal cancer. The results showed that TDs remained an independent risk factor for OS after adjusting for baseline factors (age, sex, marital status), stage, other risk factors (CEA, tumor size, tumor differentiation, PNI), treatments (postoperative RT, postoperative chemotherapy), and all the included variables (baseline, stage, treatment, and others), which are all shown in **Table 2**.

**Table 2:** Effect of pretreatment tumor deposits on overall survival in rectal cancer patients.

	HR (95% CI)	*P*-value
Unadjusted	1.89 (1.61, 2.21)	<0.001
Model 1^a^	1.89 (1.61, 2.21)	<0.001
Model 2^b^	1.74 (1.48, 2.04)	<0.001
Model 3^c^	1.61 (1.36, 1.89)	<0.001
Model 4^d^	1.86 (1.58, 2.18)	<0.001
Model 5^e^	1.57 (1.33, 1.85)	<0.001

HR, hazard ratio; CI, confidence interval; CEA, carcinoembryonic antigen; PNI, perineural invasion.

a Adjusted for baseline factors (age, sex, marital status).

b Adjusted for stage.

c Adjusted for other risk factors (CEA, tumor size, tumor differentiation, PNI).

d Adjusted for treatments (postoperative radiotherapy, postoperative chemotherapy).

e Adjusted for baseline factors, stage, other risk factors, and treatments.

### Clinicopathological Characteristics of Patients With TDs

**Supplementary Table 1** shows the baseline clinical and pathological characteristics of the training and validation sets. Apparent differences were observed between the two cohorts. Briefly, in the training set, the proportions of stage IV, tumor size >5 cm, LNR ≤0.038, LOODS ≤−1.330, PLNC = 0, and receiving postoperative RT were 29.0%, 42.4%, 61.5%, 66.4%, 41.3%, and 37.4%, respectively; in the validation cohort, the corresponding proportions were 11.8%, 16.7%, 18.6%, 14.7%, 17.6%, and 9.8%, respectively.

Among the N stage, LNR, LOODS, PLNC, and NLNC, we identified the optimal lymph node staging system using the C-index and the Akaike information criterion (AIC). The LOODS exhibited the highest C-index (0.61) and minimum AIC (2,991), compared with LNR (C-index: 0.61, AIC: 2,995), PLNC (C-index: 0.59, AIC: 3,017), NLNC (C-index: 0.58, AIC: 3,019), and N stage (C-index: 0.61, AIC: 2,995). Therefore, LOODS was included in the further analysis.

### Variable Selection

Model 1 was constructed using the variables identified from the LASSO. As shown in **Supplementary Figures 2A, B**, a coefficient profile figure was produced against the ln (*λ*) sequence. With a lambda of 0.144, the LASSO regression analysis identified the seven non-zero coefficients: age, CEA, T stage, M stage, LOODS, tumor differentiation, and postoperative chemotherapy (**Table 3**).

**Table 3:** Risk factors for rectal cancer patients with tumor deposits in the training set.

	Model 1		Model 2		Model 3	
	HR (95% CI)	*P*-value	HR (95% CI)	*P*-value	HR (95% CI)	*P*-value
Age	1.59 (1.23, 2.07)	<0.001	1.70 (1.31, 2.20)	<0.001	1.57 (1.21, 2.04)	0.001
Sex	–	–	–	–	–	–
Marital status	–	–	0.66 (0.52, 0.85)	0.001	–	–
CEA	1.87 (1.41, 2.48)	<0.001	1.80 (1.36, 2.39)	<0.001	1.97 (1.49, 2.59)	<0.001
T stage						
T3 vs. T1–2	1.19 (0.70, 2.03)	0.522	–	–	–	–
T4 vs. T1–2	1.50 (0.84, 2.69)	0.175	–	–	–	–
M stage	3.18 (2.40, 4.22)	<0.001	3.12 (2.36, 4.13)	<0.001	3.25 (2.45, 4.30)	<0.001
LOODS						
≤0.600 vs. ≤0.038	2.31 (1.49, 3.60)	<0.001	2.40 (1.54, 3.75)	<0.001	2.34 (1.51, 3.64)	<0.001
>0.600 vs. ≤0.038	3.87 (2.36, 6.33)	<0.001	4.20 (2.55, 6.91)	<0.001	3.96 (2.42, 6.48)	<0.001
Tumor size	–	–	1.25 (0.97, 1.61)	0.081	–	–
Tumor differentiation	1.70 (1.30, 2.23)	<0.001	1.63 (1.25, 2.13)	<0.001	1.80 (1.38, 2.34)	<0.001
PNI	–	–	1.34 (1.04, 1.74)	0.026	–	–
Postoperative radiotherapy	–	–	–	–	–	–
Postoperative chemotherapy	0.47 (0.35, 0.62)	<0.001	0.47 (0.36, 0.62)	<0.001	0.45 (0.34, 0.60)	<0.001

CEA, carcinoembryonic antigen; LOODS, log odds of metastatic lymph nodes; PNI, perineural invasion; HR, hazard ratio; CI, confidence interval.

Model 2 was constructed using potential factors *via* the BSR. With a minimum AIC of 2,815, nine potential factors, namely, age, marital status, CEA, M stage, LOODS, tumor size, tumor differentiation, PNI, and postoperative chemotherapy, were selected and incorporated into model 2 (**Table 3**).

Considering that we aimed to establish an accurate and convenient model for predicting OS of patients with TDs, the seven factors identified from LASSO were used in the BSR analysis (model 3). Finally, the LASSO-BSR identified the following six most powerful factors: age, CEA, M stage, LOODS, tumor differentiation, and postoperative chemotherapy. All selected variables showed significant statistical differences (all *P* < 0.05, **Table 3**), and model 3 was constructed.

The final prediction model was determined by C-index and AUC at 3, 4, and 5 years (**Table 4**). In the training set, all three models exhibited higher C-indexes and AUCs at 3, 4, and 5 years than AJCC (all *P* < 0.05), but there were no significant differences among the three models. In the validation set, only model 3 exhibited better performance than AJCC in terms of C-index and AUCs at 3, 4, and 5 years (all *P* < 0.05). Therefore, model 3 was chosen as the final prognostic model.

**Table 4:** Comparison of C-indexes and AUCs between different models.

	Training set				Validation set			
	AJCC	Model 1	Model 2	Model 3	AJCC	Model 1	Model 2	Model 3
C-index	0.65	0.76^*^	0.77^*^	0.76^*^	0.68	0.79	0.79	0.81^*^
3-year AUC	0.69	0.82^*^	0.83^*^	0.82^*^	0.72	0.85^*^	0.84^*^	0.87^*^
4-year AUC	0.69	0.83^*^	0.84^*^	0.83^*^	0.65	0.76^*^	0.74	0.79^*^
5-year AUC	0.72	0.87^*^	0.88^*^	0.86^*^	0.61	0.71	0.70	0.74^*^

AUC, area under the curve; AJCC, American Joint Committee on Cancer.

*p < 0.05 (vs. AJCC).

### Construction and Validation of the Nomogram

**Figure 2:**
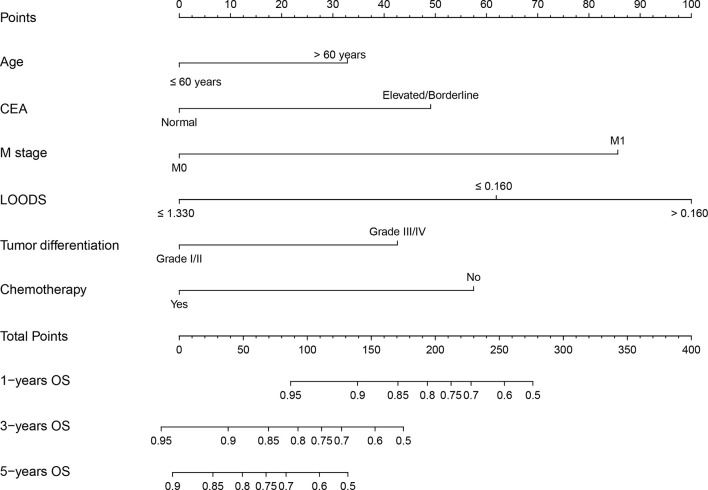
Nomogram for predicting overall survival (OS) of rectal cancer patients with tumor deposits. CEA, carcinoembryonic antigen; LOODS, log odds of metastatic lymph nodes.

A nomogram was established based on model 3 (**Figure 2**). The C-indexes of the nomogram in the training and validation sets were 0.76 [95% confidence intervals (CI) = 0.73–0.79] and 0.81 (95% CI = 0.73–0.88), respectively. Calibration plots showed better consistency between the predicted outcomes of the nomogram and the actual outcomes in terms of 3- and 5-year OS in the training set (**Supplementary Figures 3A, B** **)**. Similar results were observed in the validation set (**Supplementary Figures 3C, D** **)**.

**Figure 3:**
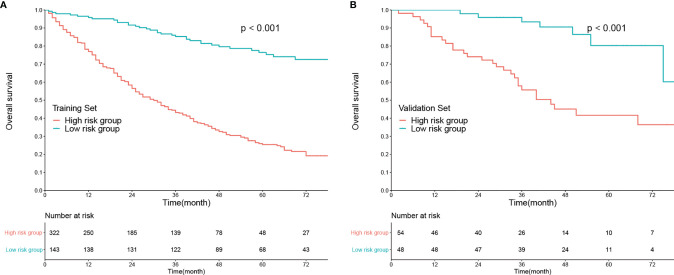
Kaplan–Meier analysis of overall survival according to risk stratification in the **(A)** training set and **(B)** validation set.

In addition, each patient received a corresponding total point according to the nomogram. The median total points were 144 (0–342) in the training set and 105 (0–330) in the validation set. A cutoff value of 100 was used to categorize the patients into two risk subgroups (low-risk and high-risk groups). K-M curves showed good predictive performance of the nomogram both in the training and validation sets (both *P* < 0.001, **Figures 3A, B** **)**.

### Clinical Applicability of the Nomogram

DCA was used to evaluate the clinical applicability of the nomogram. Compared with the eighth AJCC staging system, the DCA showed that the current nomogram had a better overall net benefit across a wide range of reasonable threshold probabilities in both the training set (**Supplementary Figure 4A**) and the validation set (**Supplementary Figure 4B**).

**Figure 4:**
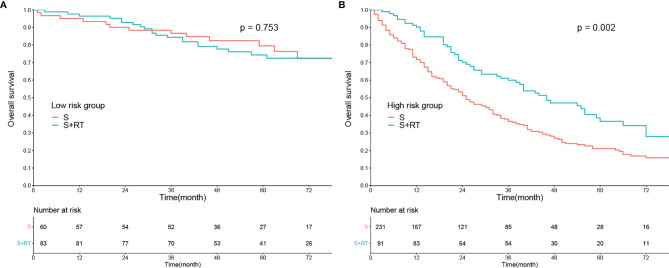
The effect of postoperative radiotherapy (RT) on overall survival of rectal cancer patients with tumor deposits. Kaplan–Meier curves in the high-risk group **(A)** and low-risk group **(B)**. S, surgery.

The current nomogram was also taken as an index to guide the management of postoperative RT. As shown in **Figure 4A**, there was no significant difference in terms of OS between subgroups receiving postoperative RT or not among low-risk patients (1-year OS: 96.38% vs. 93.33%; 3-year OS: 84.34% vs. 86.63%; 5-year OS: 74.33% vs. 79.48%; *P* > 0.05). On the contrary, among high-risk patients, survival benefit was observed between subgroups receiving postoperative RT or not (1-year OS: 90.11% vs. 71.75%; 3-year OS: 59.98% vs. 36.53%; 5-year OS: 36.57% vs. 21.07%; *P* < 0.01; **Figure 4B**).

## Discussion

Growing concerns have been raised regarding TDs with increasing detection rates (4, 7), but the clinical value of TDs has been far from being explored. To the best of our knowledge, this is the first model to predict the prognosis of rectal cancer patients with TDs. We first identified the clinical significance of TDs in a population-based analysis, and then constructed a nomogram to predict the prognosis of TD-positive rectal cancer patients, which exhibited better performance and applicability than the AJCC staging system. Furthermore, the nomogram was validated in an external cohort at our center.

There has been controversy since the introduction of TD into the AJCC staging system (5). The majority considered TDs to come from lymph nodes (20), but some regarded TDs as destructive venous invasions (2, 21) or remnants of neoadjuvant treatment (4). Hence, in this study, we excluded all patients who had received neoadjuvant treatment. Nonetheless, TDs are quite an aggressive hallmark of rectal cancer (2–4). In the present study, TDs were significantly associated with adverse prognosis in patients with rectal cancer. Compared with TD-negative rectal cancer patients, TD-positive patients typically present with adverse features, such as elevated CEA levels, advanced TNM staging, and PNI (all *P* < 0.05), as previously reported (2, 10, 22, 23). In addition, TDs were still identified as an independent risk factor for OS, even after adjusting for baseline factors, stage, other risk factors, treatments, and all the included variables in this study (all *P* < 0.05).

However, the positioning of TDs in the TNM system has always been underestimated. TDs are only embodied in N1c in the eighth AJCC staging system (6). However, the presence of TDs in LNM patients also indicates a poor prognosis (10, 11), and Mayo et al. (9) showed that TDs were associated with worse 3-year OS in patients with any known and unknown N categories, both of which indicated that the current staging system might not be enough to predict the prognosis of TD-positive patients. In this study, we established a novel prognostic nomogram for rectal cancer patients with TDs, which exhibited better predictive performance than the eighth AJCC staging system in both the training and validation sets (C-index: 0.76 vs. 0.65, *P* < 0.05, in the training set; 0.81 vs. 0.68, *P* < 0.05, in the validation set, respectively). DCA curves showed that the nomogram had better net benefits than the eighth AJCC staging system. Similar findings were confirmed in the external validation set, indicating the robustness of the nomogram.

Considering the ignorance of dissected lymph node (LND) numbers in the current N staging system, we introduced the variables of LNR, LOODS, PLN, and NLN to avoid the phenomenon of “stage migration” in case of unsatisfactory LND (24). As previously reported (25, 26), all nodal staging systems exhibited good calibration among patients with TDs (all *P* < 0.05), and LOODS were chosen as the optimal nodal staging in the current study, with the highest C-index and the minimum AIC. The reasons why LOODS were superior to others, in our opinion, might be as follows:1) LOODS took full account of PLN and NLN numbers to minimize the possibility of “staging migration” due to poor lymph node dissection; 2) LOODS could be further stratified in patients without lymph node metastasis, which would decrease with the increasing PLN number; and 3) LNR would lose the value in cases where all lymph nodes are positive, but LOODS would not (25).

To avoid overfitting or underfitting of the model, we adopted three statistical methods to select the candidate variables. As is known to all, the big disadvantage of the Cox regression analysis is its unmanageable confounders (15), which will result in overfitting of the model. LASSO regression (13, 27) can process all independent variables simultaneously and introduce the variable *λ* (lambda). With the increase in *λ*, the regression coefficient *β* of each variable decreases, and some of them turn to 0, indicating that the variable makes little contribution to the model at this time and can be eliminated. In addition, BSR (14, 28) can eliminate factors that have an impact on outcome but not that important to make the model much more practical. The combination of these two methods, LASSO followed by BSR (29), solves the collinearity between independent variables but does not weaken the predictive efficiency. Hence, in this study, we adopted LASSO followed by BSR to establish model 3, which exhibited non-inferiority in discrimination and calibration compared with models 1 and 2 based on LASSO and BSR alone, but comprised the minimum variables with wider external application, which was subsequently validated in an external set. Of note, there were apparent differences in baseline characteristics between sets of training and validation, which indicated that the current model might have universal applicability.

Postoperative management, regardless of prophylactic or salvage therapy, is also a concern in order to improve the long-term prognosis of rectal cancer. Substantial evidence has shown that TDs are correlated with increased local recurrence and distant metastasis and impaired DFS and OS (10, 11, 30). Delattre et al. (2) found that postoperative chemotherapy would benefit patients with TDs, which was also validated by Shi et al. (31). As one of the most common modalities, postoperative RT also plays an important role in the postoperative management of rectal cancer, especially for those who do not receive preoperative RT. In our previous study (32), we identified the survival benefit of postoperative RT for patients with pT3N0 disease in the high-risk subgroup. Nonetheless, it remains controversial whether all TD-positive patients should receive postoperative RT (33). In the current study, we also found that postoperative RT could only prolong the median OS of patients in the high-risk subgroup, but not in the low-risk subgroup. In summary, the current nomogram could also be used for decision-making in the management of postoperative RT.

However, this study has several limitations. First, both the training and validation sets were retrospective; therefore, the current model needs to be further validated by a prospective cohort. Second, considering that the etiology and management of rectal cancer are slightly different between the United States and China, as depicted in **Supplementary Table 1**, international multicenter cohorts are warranted to verify the performance of the current nomogram. Third, considering the uncertainty of TD origin (2, 4, 20, 21), we only included patients who did not receive neoadjuvant treatment in the present study; hence, the nomogram might not be appropriate for those receiving neoadjuvant treatment. Finally, considering that most of the TD numbers were unattainable in the SEER database and it was insufficient to regard the number of TDs as a prognostic parameter (2, 10), the variable of TD number was not considered in the present study.

## Conclusion

In conclusion, we identified a novel nomogram for predicting the prognosis of rectal cancer patients with TD. The current model could provide reliable evidence for clinical decision-making, although it still deserves further validation.

## Data Availability Statement

The dataset analyzed in this study from SEER can be obtained from: https://seer.cancer.gov/data/. Other data supporting the conclusions of this article are available from the corresponding author upon reasonable request.

## Ethics Statement

This study was conducted under the ethical guidelines of the Helsinki Declaration. We acquired approval from Fujian Cancer Hospital’s Ethics Committee (K2021-050-01), which waived back the individual informed consent owing to that the clinicopathological data were extracted retrospectively. On the other hand, we gained an official permit to access the research data from the SEER database.

## Author Contributions

XHZ, LW, LS, XQZ, LH, GC, JW contributed to conception and design. XHZ, LW conducted data collection and analyzed the data. XHZ, LW, LS, XQZ, LH, GC, JW interpreted the data. XHZ, LW, LS drafted the manuscript. XQZ, LH, GC, JW contributed critical revision of the manuscript. All authors read and approved the final manuscript.

## Funding

This research was funded by the Science and Technology Program of Fujian Province, China (Nos. 2019L3018 and 2019YZ016006); the Fujian Province Finance Department Project (No. (2019)827); the Fujian Province Natural Science Foundation (2021J01438); the Fujian Provincial Clinical Research Center for Cancer Radiotherapy and Immunotherapy (2020Y2012); the National Clinical Key Specialty Construction Program.

## Conflict of Interest

The authors declare that the research was conducted in the absence of any commercial or financial relationships that could be construed as a potential conflict of interest.

## Publisher’s Note

All claims expressed in this article are solely those of the authors and do not necessarily represent those of their affiliated organizations, or those of the publisher, the editors and the reviewers. Any product that may be evaluated in this article, or claim that may be made by its manufacturer, is not guaranteed or endorsed by the publisher.
