# Supplementary material for: Prognostic Nomogram for Rectal Cancer Patients With Tumor Deposits
Source: Front Oncol. 2022 Feb 2;12:808557. doi: 10.3389/fonc.2022.808557 (PMC8847760; doi:10.3389/fonc.2022.808557)
Supplement: Supplementary file 1 [file DataSheet_1.docx]

Supplementary Material

# Supplementary Data

## Supplementary Tables

Supplementary Table 1 Baseline clinicopathological characteristics of rectal cancer patients with tumor deposits in the training set and validation set.

|  | Training set | Validation set |
| --- | --- | --- |
|  | (N=465) | (N=102) |
| Age |  |  |
| ≤ 60 years | 231 (49.7%) | 65 (63.7%) |
| > 60 years | 234 (50.3%) | 37 (36.3%) |
| Sex |  |  |
| Male | 281 (60.4%) | 66 (64.7%) |
| Female | 184 (39.6%) | 36 (35.3%) |
| Marital status |  |  |
| Unmarried | 206 (44.3%) | 0 (0%) |
| Married | 259 (55.7%) | 102 (100%) |
| Carcinoembryonic antigen |  |  |
| Normal | 220 (47.3%) | 50 (49.0%) |
| Elevated/Borderline | 245 (52.7%) | 52 (51.0%) |
| Stage |  |  |
| III | 330 (71.0%) | 90 (88.2%) |
| IV | 135 (29.0%) | 12 (11.8%) |
| T stage |  |  |
| T1-2 | 56 (12.1%) | 8 (7.9%) |
| T3 | 313 (67.3%) | 81 (79.4%) |
| T4 | 96 (20.6%) | 13 (12.7%) |
| N stage |  |  |
| N1 | 241 (51.8%) | 59 (57.8%) |
| N2 | 224 (48.2%) | 43 (42.2%) |
| M stage |  |  |
| M0 | 330 (71.0%) | 90 (88.2%) |
| M1 | 135 (29.0%) | 12 (11.8%) |
| Tumor size |  |  |
| ≤ 5cm | 268 (57.6%) | 85 (83.3%) |
| > 5cm | 197 (42.4%) | 17 (16.7%) |
| Tumor differentiation |  |  |
| Grade I/II | 347 (74.6%) | 89 (87.3%) |
| Grade III/IV | 118 (25.4%) | 13 (12.7%) |
| Perineural invasion |  |  |
| Absent | 279 (60.0%) | 53 (52.0%) |
| Present | 186 (40.0%) | 49 (48.0%) |
| (continued on next page) | | |
| Supplementary Table 1 (Continued) | | |
|  | Training group | Validation group |
|  | (N=465) | (N=102) |
| LNR |  |  |
| ≤ 0.038 | 286 (61.5%) | 19 (18.6%) |
| ≤ 0.600 | 78 (16.8%) | 75 (73.5%) |
| > 0.600 | 101 (21.7%) | 8 (7.9%) |
| LOODS |  |  |
| ≤ -1.330 | 309 (66.4%) | 15 (14.7%) |
| ≤ 0.160 | 78 (16.8%) | 79 (77.5%) |
| > 0.160 | 101 (21.7%) | 8 (7.9%) |
| PLNC |  |  |
| 0 | 192 (41.3%) | 18 (17.6%) |
| ≤ 4 | 189 (40.6%) | 56 (54.9%) |
| > 4 | 84 (18.1%) | 28 (27.5%) |
| NLNC |  |  |
| ≤ 7 | 112 (24.1%) | 12 (11.8%) |
| > 7 | 353 (75.9%) | 90 (88.2%) |
| Postoperative radiotherapy |  |  |
| No | 291 (62.6%) | 92 (90.2%) |
| Yes | 174 (37.4%) | 10 (9.8%) |
| Postoperative chemotherapy |  |  |
| No | 125 (26.9%) | 18 (17.6%) |
| Yes | 340 (73.1%) | 84 (82.4%) |
| LND, dissected lymph nodes; LNR, lymph node ratio; LOODS, log odds of metastatic lymph nodes; PLN, positive lymph node; NLN, negative lymph node | | |

## Supplementary Figures


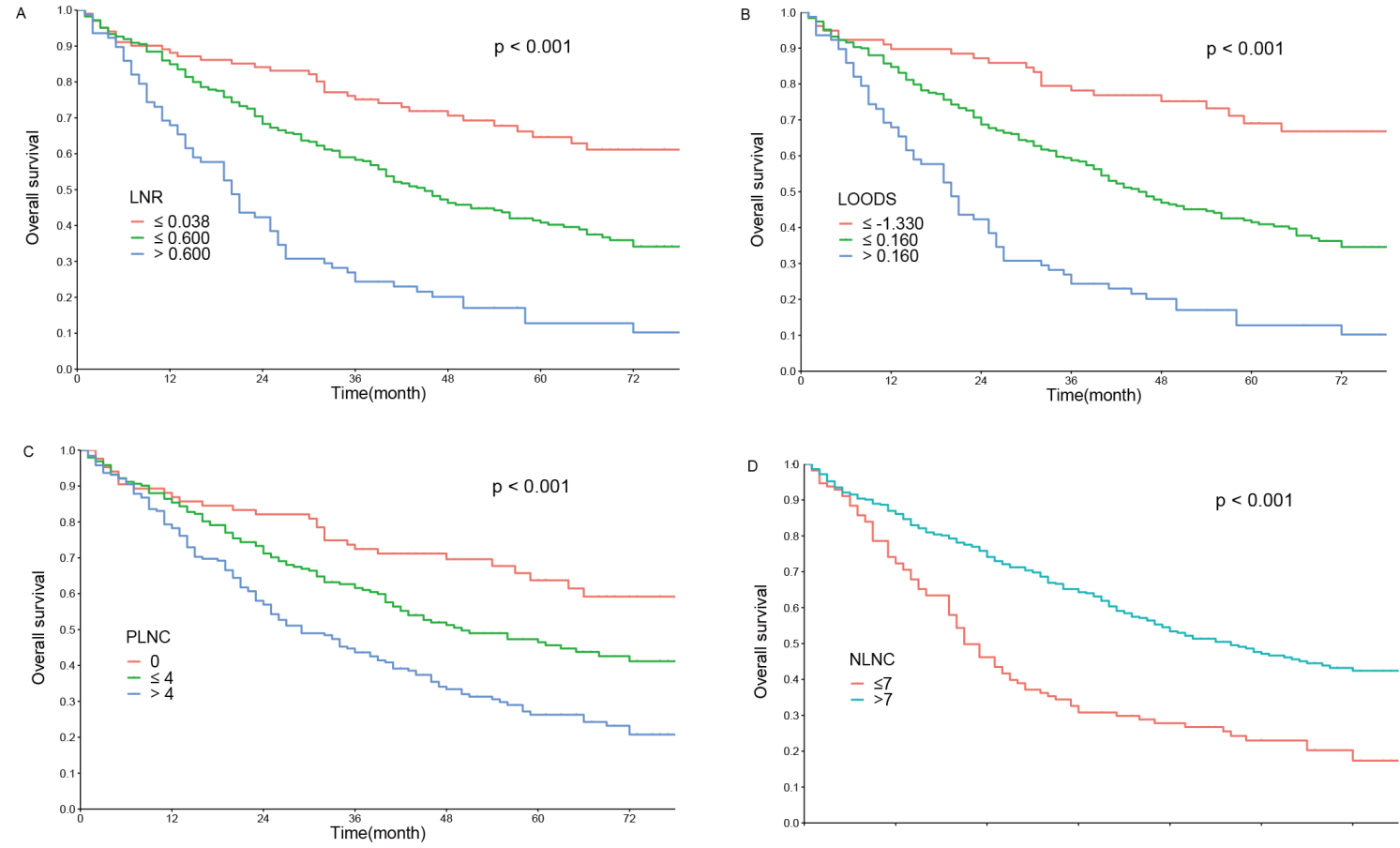


**Supplementary Figure 1.** Overall survival according to different lymph node staging systems. LNR, lymph node ratio; LOODS, log odds of metastatic lymph nodes; PLNC, positive lymph node; NLNC, negative lymph node.


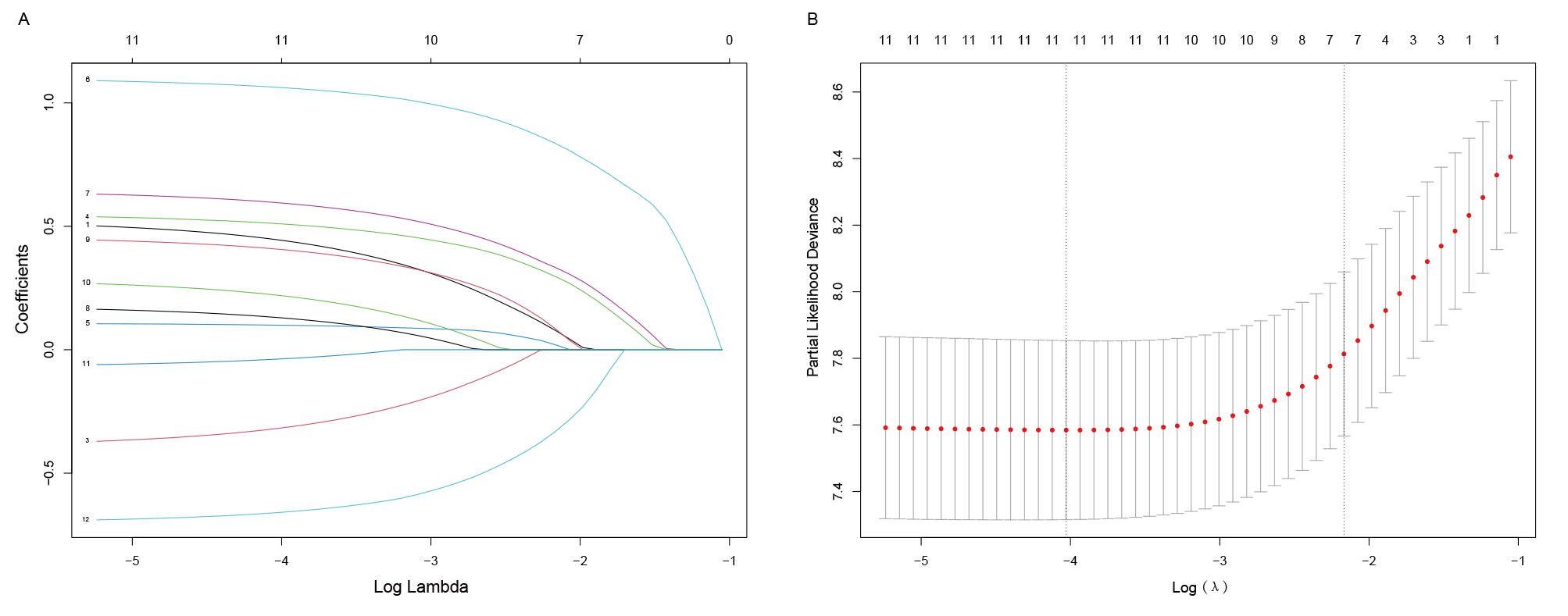


**Supplementary Figure 2.** Potential variable selection using LASSO regression model. (A) LASSO coefficient profiles of the 12 variables. (B) The tuning parameter plot. The x-axis represents log-transformed lambda values, and the y-axis represents the partial likelihood deviance. The vertical dashed line indicates the minimal partial likelihood deviance. LASSO, least absolute shrinkage and selection operator regression.


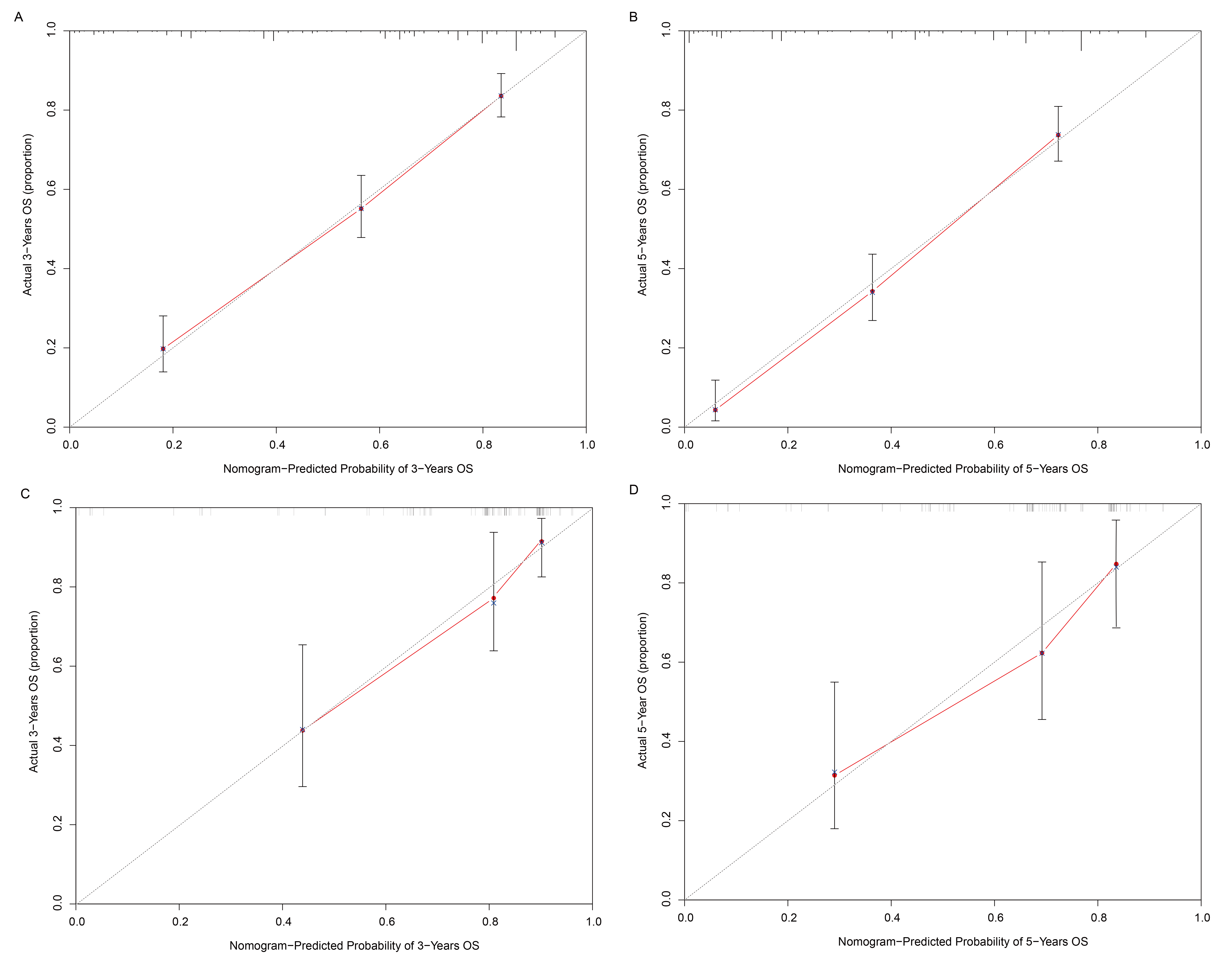


**Supplementary Figure 3.** The calibration curve for predicting patient’s overall survival (OS) at (A) 3-years and (B)5-years in the training set and (C) 3-years and (D) 5-years in the validation set.


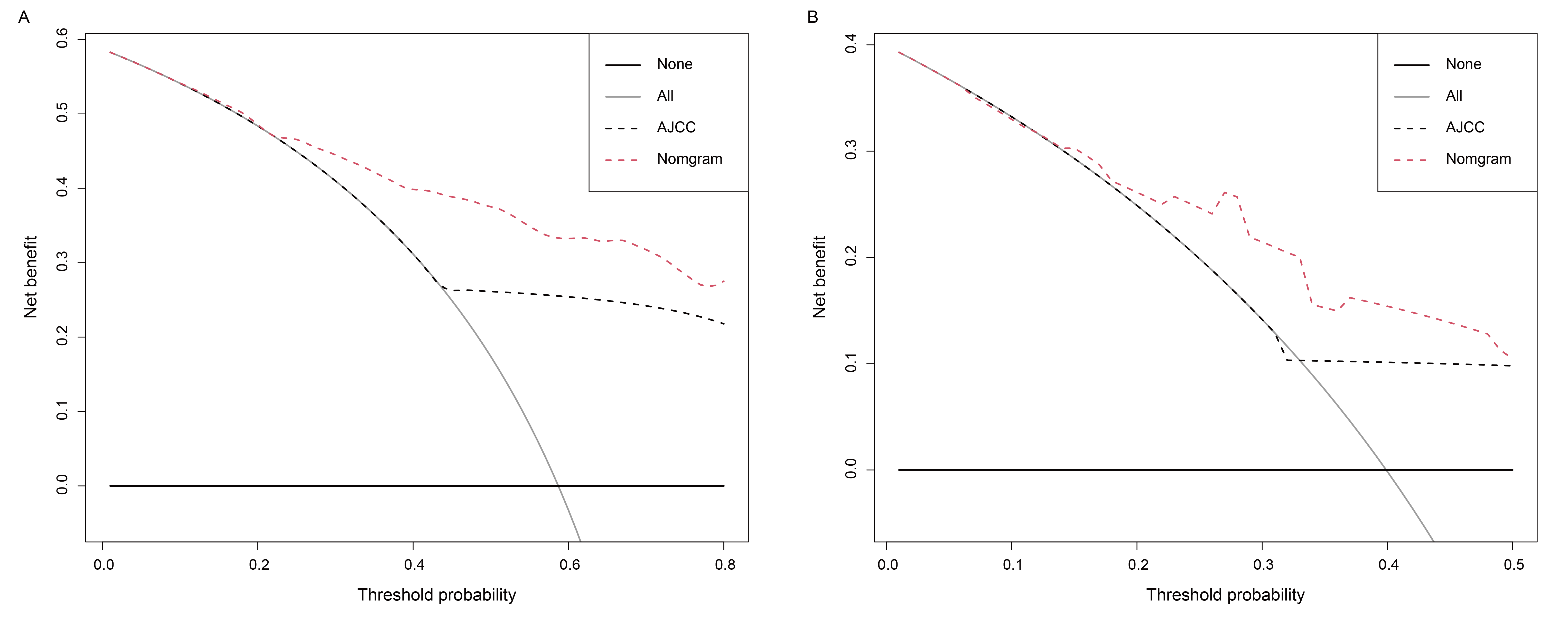
**Supplementary Figure 4.** Decision curve analysis for the Nomogram and AJCC stage in prediction of prognosis of rectal cancer with tumor deposits in the (A) training set and (B) validation set. AJCC, American Joint Committee on Cancer.
